# Noninvasive Monitoring of Hepatic Damage from Hepatitis C Virus Infection

**DOI:** 10.1155/2011/325470

**Published:** 2011-02-06

**Authors:** J. Alavez-Ramírez, J. L. Fuentes-Allen, J. López-Estrada

**Affiliations:** ^1^División Académica de Ciencias Básicas, Universidad Juárez Autónoma de Tabasco, Cunduacán, 86690 México, TAB, Mexico; ^2^Hospital de Infectología, Centro Médico Nacional la Raza, Instituto Mexicano del Seguro Social, 01200 México, DF, Mexico; ^3^Departamento de Matemáticas, Facultad de Ciencias, Universidad Nacional Autónoma de México, 04510 México, DF, Mexico

## Abstract

The mathematical model for the dynamics of the hepatitis C proposed in Avendaño et al. (2002), with four populations (healthy and unhealthy hepatocytes, the viral load of the hepatitis C virus, and T killer cells), is revised. Showing that the reduced model obtained by considering only the first three of these populations, known as basic model, has two possible equilibrium states: the uninfected one where viruses are not present in the individual, and the endemic one where viruses and infected cells are present. A threshold parameter (the basic reproductive virus number) is introduced, and in terms of it, the global stability of both two possible equilibrium states is established. Other central result consists in showing, by model numerical simulations, the feasibility of monitoring liver damage caused by HCV, avoiding unnecessary biopsies and the undesirable related inconveniences/imponderables to the patient; another result gives a mathematical modelling basis to recently developed techniques for the disease assessment based essentially on viral load measurements.

## 1. Introduction

Hepatitis C virus (HCV) infection represents a serious problem of public health with strong clinical and economic repercussions. Lethal consequences may arise from a subclinical acute infection followed by a latent period, and eventually hepatic cirrhosis (from 20% to 30% of the cases) or to hepatocellular carcinoma (with a far smaller percentage) [1], as final events at the end stage of chronic liver disease. It was not before 1989, that the infectious viral agent was identified as HCV in patients with hepatitis not A and not B [2]. At present, six different genotypes of HCV have been identified with diverse biological and clinical behaviors. For instance, it has been observed that genotype 1 response to therapy is less effective than one by genotypes 2 and 3 [3].

The most frequent ways for HCV transmission are blood transfusion, use of intravenous drugs, hemodialysis, tattoos, high-risk sexual behavior, occupational exposition of medical and paramedical personnel, vertical transmission from mother to her product, and organ transplants from an infected donor. It is important to say that the mechanism for HCV transmission is unidentified in a high percentage of patients (from 20% to 40%) [4].

The incubation period of HCV is 50 days in average, ranging from 15 to 150 days [2]. Factors influencing the rate of progression from chronic hepatitis to cirrhosis appear to include age at time of exposure, duration of infection, degree of previous liver damage, immunological system status, and HCV genotype. The disease progression is insidious; the clinically significant time of evolution varies: the diagnosis of chronic hepatitis, cirrhosis, and hepatocellular carcinoma have been estimated to be 10, 20, and 30 years, respectively [1, 5]. The majority of patients show increased levels of aminotransferases as well as hepatocellular damage. Bleeding of esophageal varices, ascitis, coagulopathy, and encephalopathy, among others, may be observed at advanced stages of the evolution. The progression of the disease is variable, not always orderly nor sequential. Patients can evolve from chronic hepatitis directly to hepatocellular carcinoma without first developing cirrhosis, especially those with genotype 1b [5].

The mechanisms of replication and persistence of the HCV at the cellular level have not been completely characterized yet. Nevertheless, it is well known that it takes place at hepatic level, and no replication at extrahepatic sites has been reported up to date. Due to the high mutation rate of HCV, a great amount of different immunological variants appear; this variance partly explains the virus ability to evade the host's immunological control, and the infection eventually becomes a chronic disease in most cases. Furthermore, the strong mutagenesis of the virus makes it very difficult to develop an effective vaccine.

Nowadays, chronic hepatitis C therapy approved by both, the Food and Drug Administration (FDA) and the European Medicines Agency (EMEA), consists of the administration of *α* − 2*a* or *α* − 2*b* pegylated interferon plus ribavirin [6, 7]. It is important to observe that central goal of the treatment is to substantially decrease the viral load [8–10].

The treatment for an HCV-infected patient essentially depends on the degree of his/her hepatic damage. Percutaneous liver biopsy is an invasive tool that has been extensively used to assess the degree of hepatic damage, despite having serious inconveniences. This poses a relevant problem with significant impact on medicine to propose a noninvasive procedure for monitoring the hepatic damage.

In the next section we discuss the use, importance, and inconveniences of the percutaneous liver biopsy. In Section 3 we present a model of four populations (healthy and unhealthy hepatocyte, viral load, and *T*-killer cells), originally proposed by Avendaño et al. [11]. In Section 4, following Avendaño, we develop the qualitative analysis of the reduced model to the first three populations above mentioned. In Section 5, we show that the evolution of healthy and unhealthy hepatocyte populations and viral load for both models of three and four populations, is practically the same. In Section 6, we present the main result of this research. We show that numerical estimation of parameters in the reduced model for hepatitis C disease dynamics, using only a sufficient number of viral load measurements and a reasonable proposal for the initial value for populations, provides us the bases for a noninvasive technique to asses the hepatic damage. Finally, in Section 7 we discuss the results and theirs implications.

## 2. Liver Biopsies and Motivation for an Alternative

The clinical study of a patient starts when his/her infection status is detected by using a serological HCV antibodies test. In HCV positive patient, viral load should be quantified in order to establish the intensity of viral replication. Then identification of the HCV genotype is performed by molecular procedures in those patients with detectable viral load; this is necessary to define duration of the therapy and for prognostic purposes. Finally, liver biopsy is done, usually by a percutaneous puncture, to measure the degree and extent of liver tissue damage.

Percutaneous liver biopsy is an invasive method that had been used extensively to evaluate the degree (intensity of necroinflammatory activity), and the stage (extent of fibrosis or the presence of cirrhosis) of hepatic injury. This method consists in the extraction of a small piece of hepatic tissue by the insertion of a needle into the liver, which provides useful information to classify the patient according to the stage of the disease. Hepatic biopsy had been considered the best available tool for diagnosing and evaluating the treatment efficacy [12]; however, it could be risky, and even produce pain and temporal disability to the patient [13]. On the other hand, since tissue samples obtained by this method are very small, it is debatable if they are representative of the whole liver status [14–16].

Due to its inconsistencies and inconveniences (some serious), the usefulness of the liver biopsy is presently considered less important than before; some of its questionable points are as follows. (i) *Tissue representativeness:* are tissue samples obtained by percutaneous liver biopsies really representative of the whole liver? (ii) *Finding reproductiveness.* The findings by different pathologists or from different samples could vary remarkably either in minor or major degree, and such differences seem to be the rule, not the exception. (iii) *Biopsy usefulness:* the most important point is that biopsies were considered useful in classifying patients according to the stage of the disease, and identifying patients that had already developed cirrhosis. In both proposals, biopsies do not seem to be really useful at all. (iv) *Biopsy futility:* given the satisfactory response to therapy in patients infected by genotypes 2 or 3, biopsy is considered unnecessary. With regard to genotype 1 or 4, who only responds in 50% of the cases, performing a biopsy is still under debate [3].

The fear, pain, and the temporary disability of the patient, are considered as serious and negative aspects. Nonetheless, liver biopsy is still mandatory to assess the stage and degree of liver disease. Alternatively, nowadays we dispose of a new method to evaluate the status of the liver tissue, in particular the stage of fibrosis, named elastography (Fibroscan), which has only recently been introduced in clinical practice and is not yet available in low income countries/areas.

In addition to the panel of blood markers, are in progress another noninvasive tool for the evaluation of the extent of fibrosis [17]. These markers are useful for establishing the two ends of fibrosis spectrum (minimal fibrosis and cirrhosis) but are less helpful in assessing its mid ranges. In particular, the elastography is improved when it is combined with markers (for details, see [3]).

In the last years, the viral load count has been used as a noninvasive technique that provides useful information on the intensity of the viral replication, making unnecessary the performance of liver biopsy depending on its viral genotype [3]. This technique is very reliable and also has been used to compare the infection degree before and after a particular treatment has been decided.

## 3. Dynamics of the Hepatitis C: Model I

In this section, for completeness, we present a brief recapitulation of the original model for the dynamics of the hepatitis C proposed in [11], with special attention on those aspects with relevance to our research objectives. The model is given by the following system of ordinary differential equations (ODE):

(1) $$\begin{array}{c} \dot{H_{s}}=\beta_{s}-kH_{s}V-\mu_{s}H_{s},\\ \dot{H_{i}}=kH_{s}V-\delta H_{i}T-\mu_{i}H_{i},\\ \dot{V}=pH_{i}-\mu_{V}V,\\ \dot{T}=\beta_{T}\left(1-\frac{T}{T_{\max\;}}\right)V-\mu_{T}T, \end{array}$$

where *H*_*s*_(*t*) is the healthy liver cell population at time *t*, assuming that these cells are reproduced at the constant rate *β*_*s*_ and die with a per capita rate *μ*_*s*_, whereas *H*_*i*_(*t*) is the infected liver cell population at time *t*. The healthy liver cells are infected at a rate proportional to the product of *H*_*s*_ and *V*, with a proportionality constant *k*, and the infected ones dying with a per capita rate *μ*_*i*_. *V*(*t*) is the HCV viral load at time *t*. Hepatitis C virions are produced by the infected cells at rate of *p* virions per infected cell per day. On the other hand, viruses die with a per capita constant rate *μ*_*V*_. *T*(*t*) is the population of the *T* killer cells (CD8^+^ cytotoxic cells) at time *t*. These cells kill infected ones at a rate proportional to the product of *H*_*i*_ and *T*, with a proportionality constant *δ*. In the presence of HCV, the *T* killer cells reproduction is proportional to the viral load *V* with a saturation rate *β*_*T*_(1 − *T*/*T*_max_), where *β*_*T*_ is the *T* cell growth rate, and *T*_max_ is the possible maximum level of the *T* cell population. Furthermore, *T* cells die at a per capita constant rate *μ*_*T*_.

Note the region

(2) $$\begin{array}{cc} \Omega & =\left\{\left(H_{s},H_{i},V,T\right)\in {\mathbb{R}}_{+}^{4}|\begin{array}{c} 0\leq H_{s}\leq H_{M},\;0\leq H_{i}\leq H_{M}\\ H_{s}+H_{i}\leq H_{M},\;0\leq V\leq V_{M}\\ 0\leq T\leq T_{M} \end{array}\right\} \end{array},$$

where *H*_*M*_ = *β*_*s*_/*μ*_*s*_, *V*_*M*_ = (*p*/*μ*_*V*_)*H*_*M*_, *T*_*M*_ = (*β*_*T*_/*μ*_T_*)*V*_*M*_, and *μ*_T_* = *μ*_*T*_ + (*β*_*T*_/*T*_max_)*V*_*M*_ is positively invariant subset for system (1). That is, every solution path of this system with initial conditions in Ω will remain in Ω for all future time.

The value of the threshold parameter

(3) $$\begin{array}{c} R_{0}=\frac{kp\beta_{s}}{\mu_{i}\mu_{s}\mu_{V}} \end{array}$$

which is named *basic reproductive virus number*, plays a central role in the analysis of qualitative global behavior of solutions of the system (1) (i.e., the disease evolution to the cure, or either to the chronic illness), clearly with relevant implications for the treatment of the hepatitis C.

The central results are as follows.

1. If *R*_0_ ≤ 1, then the system (1) has one only admissible equilibrium state in Ω, the trivial one *I*_0_ = (*β*_*s*_/*μ*_*s*_, 0,0, 0), which is globally asymptotically stable. The model predicts that without importing the intensity of the infection (i.e., except that the value of *V*_0_ ≤ *V*_*M*_), the infected individual, eventually, always will be healthy.
2. If *R*_0_ > 1, then the system (1) has two admissible equilibrium states:
  - the trivial one *I*_0_ = (*β*_*s*_/*μ*_*s*_, 0,0, 0), which is now unstable;
  - the endemic one (V* > 0)
    (4) $$\begin{array}{c} I_{1}=\left(\frac{\beta_{s}}{kV^{*}+\mu_{s}},\frac{\mu_{V}V^{*}}{p},V^{*},\frac{\beta_{T}T_{\max\;}V^{*}}{\beta_{T}V^{*}+\mu_{T}T_{\max\;}}\right) \end{array}$$
    which is globally asymptotically stable and that corresponds to the endemic patient of hepatitis C. Furthermore, *I*_1_ ∈ int (Ω) if *μ*_*i*_ > *μ*_*s*_.

Finally, it is very important to observe that the parameters related to the immune response (i.e., to *T* killer cells) are not present in the threshold parameter *R*_0_. For this reason, in the following we restrain our study to basic model with only three populations (healthy and unhealthy hepatocytes, and the viral load of the HCV).

## 4. Dynamics of the Hepatitis C: Reduced Model

As it was just mentioned above, only six of the ten parameters in the model (1) are present in the threshold parameter *R*_0_. On the other hand, it is well known that immunological response, in principle, is inefficient in the presence of HCV infection. Then, in the following, *T* killer cell population will not be considered. So, the model (1) is reduced to the following one:

(5) $$\begin{array}{c} \dot{H_{s}}=\beta_{s}-kH_{s}V-\mu_{s}H_{s},\\ \dot{H_{e}}=kH_{s}V-\mu_{e}H_{e},\\ \dot{V}=pH_{e}-\mu_{V}V. \end{array}$$

This basic model for that hepatitis C dynamics has been reported by [9, 18–21], among others. All parameters in the model are positive. It is a simple matter to verify that any initial value problem for the differential equations system (5) satisfy the locally existence and uniqueness theorem conditions.

**Figure 1:**
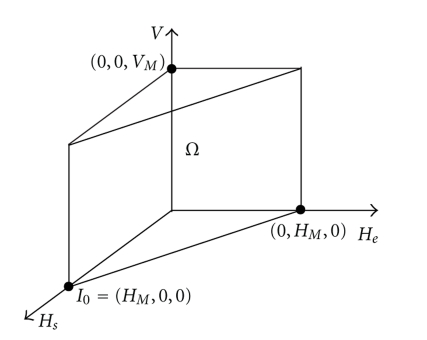
Set Ω is positive invariant.

As in [11], we begin with regarding the set

(6) $$\Omega =\left\{\left(H_{s},H_{e},V\right)\in {\mathbb{R}}_{+}^{3}|\begin{array}{c} 0\leq H_{s}\leq H_{M},\;0\leq H_{e}\leq H_{M},\\ H_{s}+H_{e}\leq H_{M},\;0\leq V\leq V_{M} \end{array}\right\},$$

where *H*_*M*_ = *β*_*s*_/*μ*_*s*_ and *V*_*M*_ = (*p*/*μ*_*V*_)*H*_*M*_ (see Figure 1). Here, *H*_*M*_ is the possible maximum size of the population of healthy hepatocyte in the liver of a healthy individual, and *V*_*M*_ is the virion maximum quantity produced by all hepatocytes during their whole lifespan (i.e., it is the viral maximum load that can be support by an individual).

**Lemma 1**

If *μ*_*e*_ ≥ *μ*_*s*_, then Ω is a positive invariant subset of ℝ_+_^3^ for the system (5).

**Proof**

It is direct to verify that the vector field defined by the system (5) does not point to the exterior of Ω, on its boundary ∂Ω.

### 4.1. Equilibrium States

In this section, we show that the system (5) has, at most, two possible equilibrium states. One of them has no viruses present and corresponds to the uninfected equilibrium state (i.e., to the healthy individual), and another one has a positive constant virus load and corresponds to the endemically infected equilibrium state (i.e., to the chronic illness).

The equilibrium states of the system (5) are obtained for solving the algebraic equation system:

(7) $$\begin{array}{c} 0=\beta_{s}-kH_{s}V-\mu_{s}H_{s},\\ 0=kH_{s}V-\mu_{e}H_{e},\\ 0=pH_{e}-\mu_{V}V. \end{array}$$

For a given V*, from the third equation of (7), it follows that

(8) $$\begin{array}{c} H_{e}^{*}=\frac{\mu_{V}}{p}V^{*}. \end{array}$$

And from the first equation of (7), we have

(9) $$\begin{array}{c} H_{s}^{*}=\frac{\beta_{s}}{\mu_{s}+kV^{*}}. \end{array}$$

Substituting (9) and (8) in the second equation of (7), we obtain

(10) $$\begin{array}{c} \left(pk\beta_{s}-\mu_{s}\mu_{e}\mu_{V}-k\mu_{e}\mu_{V}V^{*}\right)V^{*}=0. \end{array}$$

If V* = 0, from (8) and (9), then it follows that *H*_e_* = 0 and *H*_s_* = *β*_*s*_/*μ*_*s*_. Therefore,

(11) $$\begin{array}{c} I_{0}=\left(\frac{\beta_{s}}{\mu_{s}},0,0\right) \end{array}$$

is a state of equilibrium of (5). This state corresponds to the healthy or not infected individual. In consequence, all the hepatic cells are healthy, and *H*_s_* = *β*_*s*_/*μ*_*s*_ is the average maximum number of cells in the liver of a healthy individual.

For V* > 0, from (10), one obtains that:

(12) $$\begin{array}{c} V^{*}=\frac{\mu_{s}}{k}\left(R_{0}-1\right), \end{array}$$

where

(13) $$\begin{array}{c} R_{0}=\frac{kp\beta_{s}}{\mu_{e}\mu_{s}\mu_{V}} \end{array}$$

is the same threshold parameter introduced in [11].

 Obviously, V* > 0 if and only if *R*_0_ > 1.

Substituting V* given by (12) in (8) and (9), it follows that

(14) $$\begin{array}{c} H_{e}^{*}=\frac{\beta_{s}}{\mu_{e}R_{0}}\left(R_{0}-1\right),\;\;H_{s}^{*}=\frac{\beta_{s}}{\mu_{s}R_{0}}. \end{array}$$

Therefore, the second equilibrium state of the system (5) is

(15) $$\begin{array}{c} I_{1}=\left(\frac{\beta_{s}}{\mu_{s}R_{0}},\frac{\beta_{s}}{\mu_{e}R_{0}}\left(R_{0}-1\right),\frac{\mu_{s}}{k}\left(R_{0}-1\right)\right) \end{array}$$

which is the equilibrium state corresponding to the endemic patient, if and only if *R*_0_ > 1.

**Theorem 2**

Assuming that *μ*_*e*_ ≥ *μ*_*s*_:

- if *R*_0_ ≤ 1, then *I*_0_ is the only equilibrium state in Ω,
- if *R*_0_ > 1, then the system (5) has two equilibrium points in Ω: The trivial *I*_0_ and the endemically infected state *I*_1_.

**Proof**

Obviously if *R*_0_ = 1 then *I*_1_ reduces to *I*_0_. And if *R*_0_ < 1, then *I*_1_ ∉ Ω. So that *I*_0_ is the only one equilibrium state in Ω. Now, if *μ*_*e*_ = *μ*_*s*_ then *H*_s_* + *H*_e_* = *H*_*M*_, and if *μ*_*e*_ > *μ*_*s*_ then 0 < *μ*_*s*_/*μ*_*e*_ < 1, and consequently we have *H*_s_* + *H*_e_* < *H*_*M*_. In any case, *H*_s_* + *H*_e_* ≤ *H*_*M*_. So, since *V*_*M*_ = (*p*/*μ*_*V*_)*H*_*M*_ and *R*_0_ > 1, it follow that V* < *V*_*M*_.

### 4.2. Stability Analysis for Equilibrium States

In the following, we study the stability properties of the equilibrium states corresponding to the *healthy* individual and the *endemically* infected patient.

#### 4.2.1. Stability of *I*_0_

The local stability of the equilibrium state *I*_0_ is determined by the eigenvalues of the matrix

(16) $$\begin{array}{c} J\left(I_{0}\right)=\left(\begin{array}{ccc} -\mu_{s} & 0 & -k\frac{\beta_{s}}{\mu_{s}}\\ 0 & -\mu_{e} & k\frac{\beta_{s}}{\mu_{s}}\\ 0 & p & -\mu_{V} \end{array}\right) \end{array}$$

which directly shows that *I*_0_ is locally asymptotically stable if and only if *R*_0_ < 1.

To prove that *I*_0_ is globally asymptotically stable in Ω, for *R*_0_ ≤ 1, we use the next La Salle's theorem [22]: if *f* : Ω → ℝ^*n*^ is continuous and locally Lipschitz, Ω ∈ ℝ^*n*^ open, and if *U* : Ω → ℝ is such that *U* ∈ *C* ^1^(Ω), *U* ≥ 0 in Ω and its derivative $\dot{U}\leq 0$ along solution paths of $\dot{y}=f(y)$ in Ω; then the set *ω*-limit, if it exists, of every solution path of $\dot{y}=f(y)$ is contained in the set $\Omega_{0}=\{y\in \Omega :\dot{U}(y)=0\}$.

Now, as in [11], we consider for system (5) the following Lyapunov-La Salle function *U* : Ω → ℝ_+_, given by

(17) $$\begin{array}{c} U\left(H_{s},H_{e},V\right)=pH_{e}+\mu_{e}V. \end{array}$$

It is clear that *U* ∈ *C* ^1^(Ω) and *U*(*H*_*s*_, *H*_*e*_, *V*) ≥ 0, for every (*H*_*s*_, *H*_*e*_, *V*) ∈ Ω. And it is directly seen that, if *R*_0_ ≤ 1, the derivative of *U* is non negative along the solution paths of system (5) in Ω. So, by La Salle's theorem, the *ω*-limit set for every solution path of system (5) with initial conditions in int (Ω) is a subset of

(18) $$\begin{array}{c} \Omega_{0}=\left\{\left(H_{s},H_{e},V\right)\in \Omega :\dot{U}\left(H_{s},H_{e},V\right)=0\right\}. \end{array}$$

**Affirmation**

If *R*_0_ ≤ 1, every solution path of system (5) with initial conditions in Ω_0_ converges asymptotically to the trivial equilibrium state *I*_0_.

The following has been proved.

**Theorem 3**

If *μ*_*e*_ ≥ *μ*_*s*_ and *R*_0_ ≤ 1, then *I*_0_ is Ω-globally asymptotically equilibrium state.

#### 4.2.2. Stability of *I*_1_

The Jacobian matrix of the endemically infected equilibrium state *I*_1_ is

(19) $$\begin{array}{c} J\left(I_{1}\right)=\left(\begin{array}{ccc} -\mu_{s}R_{0} & 0 & -\frac{k\beta_{s}}{\mu_{s}R_{0}}\\ \mu_{s}\left(R_{0}-1\right) & -\mu_{e} & \frac{k\beta_{s}}{\mu_{s}R_{0}}\\ 0 & p & -\mu_{V} \end{array}\right) \end{array}$$

and its associated characteristic polynomial is

(20) $$\begin{array}{c} p\left(\lambda \right)=\lambda^{3}+\left(a+b\right)\lambda^{2}+ab\lambda +c \end{array}$$

with

(21) $$\begin{array}{c} a=\mu_{s}R_{0}>0,\\ b=\mu_{e}+\mu_{V}>0,\\ c=\mu_{e}\mu_{s}\mu_{V}\left(R_{0}-1\right). \end{array}$$

Using the Routh-Hurwitz criterion [23], the local stability of the endemic equilibrium *I*_1_ is established. In fact, note that

(22) $$\begin{array}{c} \Delta_{1}=\det\;\left(a+b\right)=a+b>0,\\ \Delta_{2}=\det\;\left(\begin{array}{cc} a+b & 1\\ c & ab \end{array}\right)=\left(a+b\right)ab-c,\\ \Delta_{3}=\det\;\left(\begin{array}{ccc} a+b & 1 & 0\\ c & ab & a+b\\ 0 & 0 & c \end{array}\right)=c\Delta_{2}>0. \end{array}$$

From (21), if *R*_0_ > 1 then *c* > 0. Furthermore, we also have that

(23) $$\begin{array}{cc} \Delta_{2} & =\left(\mu_{s}R_{0}+\mu_{e}+\mu_{V}\right)\mu_{s}R_{0}\left(\mu_{e}+\mu_{V}\right)-\mu_{e}\mu_{s}\mu_{V}\left(R_{0}-1\right)\\ & =\left(\mu_{s}R_{0}+\mu_{e}\right)\left(\mu_{e}+\mu_{V}\right)\mu_{s}R_{0}+\mu_{s}\mu_{V}^{2}R_{0}+\mu_{e}\mu_{s}\mu_{V}>0. \end{array}$$

To prove the global asymptotic stability of *I*_1_ in Ω, we use again the La Salle's theorem, which was enunciated in the preceding subsection. But now, following [24], we start considering the following Lyapunov-La Salle function *U* : Ω → ℝ_+_:

(24) $$\begin{array}{cc} U\left(H_{s},H_{e},V\right) & =H_{s}-H_{s}^{*}\ln\;\frac{H_{s}}{H_{s}^{*}}+H_{e}-H_{e}^{*}\ln\;\frac{H_{e}}{H_{e}^{*}}\\ & \;+\frac{\mu_{e}}{p}\left(V-V^{*}\ln\;\frac{V}{V^{*}}\right). \end{array}$$

Clearly *U* ∈ *C* ^1^(Ω), and *U*(*H*_*s*_, *H*_*e*_, *V*) ≥ 0 for every *H*_*s*_, *H*_*e*_, *V* > 0. Furthermore, if *R*_0_ > 1, one can check that $\dot{U}$ is non-negative in int (Ω) (for details see [24]). Then, by La Salle's theorem, the *ω*-limit set for every solution path of system (5) with initial conditions in int (Ω) is a subset of

(25) $$\begin{array}{cc} \Omega_{0} & =\left\{\left(H_{s},H_{e},V\right)\in \mathrm{int}\;\left(\Omega \right):\dot{U}\left(H_{s},H_{e},V\right)=0\right\}\\ & \begin{array}{c} =\left\{\left(H_{s}^{*},H_{e}^{*},V^{*}\right)\right\} \end{array}. \end{array}$$

In conclusion, we have the following.

**Theorem 4**

Assuming that *μ*_*e*_ ≥ *μ*_*s*_, if *R*_0_ > 1 then *I*_1_ is globally asymptotically equilibrium state, and *I*_0_ is now an hyperbolic equilibrium state.

## 5. Numerical Comparison between the Models

In this section, the numerical results reported in [11] are compared with corresponding ones given by the reduced model studied previously in the last section. In this comparison we use the same initial condition and parameter values used in [9, 11, 18, 25, 26]. Other numerical simulations have been carried out and reported in [25–27]. In relation with initial populations, it is well known that *H*_*M*_ = 5000 cells/mm^3^ is mean hepatocyte population for a healthy individual, that it is reasonable to consider that 10% of hepatic cells are initially infected, so *H*_*s*0_ = 4500 cells/mm^3^, and *H*_*e*0_ = 500 cells/mm^3^. And *V*_0_ = 400 UI/*μ*L and *T*_0_ = 100 cells/mm^3^ are also reasonable values for a low-infection case.

The initial conditions are

(26) $$\begin{array}{c} y_{0}=\left(4500,500,400,100\right). \end{array}$$

The admissible parameter vector of the model (1) are taken as

(27) $$\begin{array}{c} \theta =\left(\beta_{s},k,\mu_{s},\mu_{e},p,\mu_{V},\delta ,\beta_{T},\mu_{T},T_{\max\;}\right). \end{array}$$

**Case 1 (cure)**

**Figure 2:**
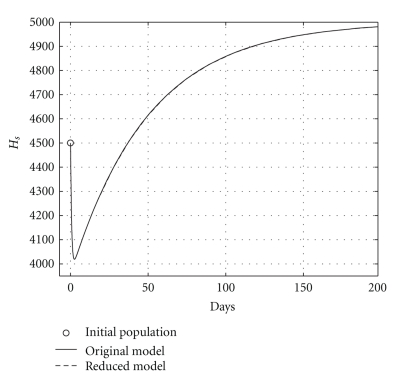
Comparing health hepatocyte populations: cured case (*R*_0_ = 0.6).

**Figure 3:**
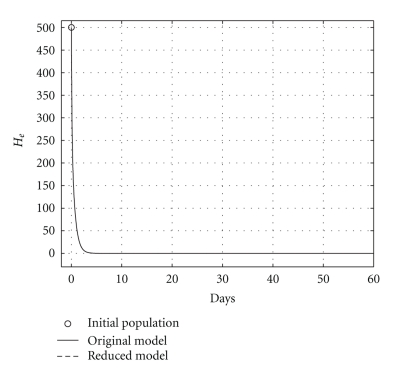
Comparing infected hepatocyte populations: cured case (*R*_0_ = 0.6).

**Figure 4:**
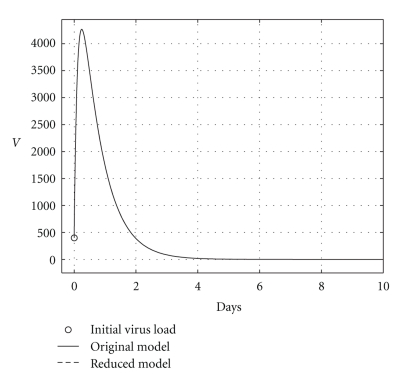
Comparing the HCV loads when the cure takes place (*R*_0_ = 0.6).

To compare numerically the evolution behavior of solutions obtained by both models under discussion, we use the following parameter values:

(28) $$\begin{array}{cc} \theta^{*} & =(100,3\times 10^{-5},2\times 10^{-2},5,100,5,10^{-5},\;\\ & \;\;3\times 10^{-4},2\times 10^{-2},1500) \end{array}$$

over a 200-day period, having that *R*_0_ = 0.6. The mortality per capita rate is given in day^−1^. Figures 2, 3, and 4, show graphically the numerical results. Clearly, the temporal courses of healthy and infected hepatocytes, and viral load are practically the same in both models.

**Case 2 (endemic disease)**

**Figure 5:**
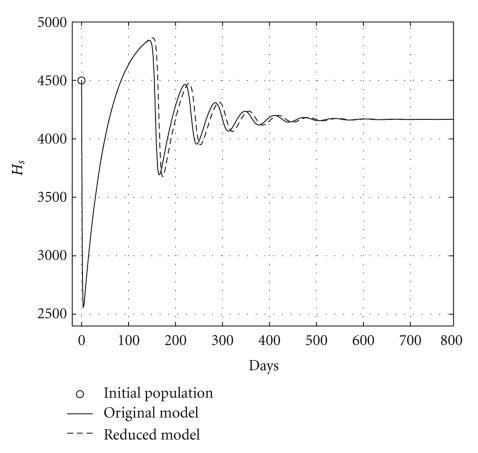
Comparing health hepatocyte evolutions: endemic case (*R*_0_ = 1.2).

**Figure 6:**
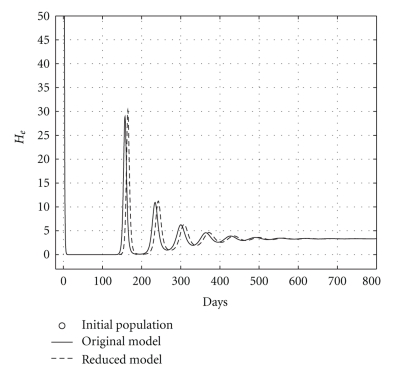
Comparing sicks hepatocyte evolutions: endemic case (*R*_0_ = 1.2).

**Figure 7:**
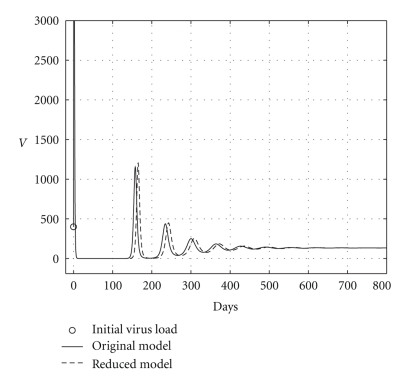
Comparing HCV loads: endemic case (*R*_0_ = 1.2).

In this case, we use the same initial conditions (26), and the same parameter vectors θ*, but now with *p* = 200, and over a 800-day period. Now, we have that *R*_0_ = 1.2. The results are graphically shown in Figures 5, 6, and 7. As it could be observed, there are some small differences between the evolution of the each three populations obtained with both models. However, they have the same asymptotic behavior.

Resuming, the evolution of the three populations under analysis are essentially the same for both models. Therefore, for the main objective of this research (the monitoring hepatic damage without biopsies), it is enough to consider the restricted model with only three populations (5).

## 6. Parameter Numerical Estimation and Hepatic Damage Monitoring without Liver Biopsies

In the following, by numerical simulations, we show that is possible to monitor the hepatic damage without biopsies, in both the cured and endemic cases. To this goal, it is indispensable to have a sufficient number of viral load measurements, and a reasonable evaluation of population initial values. These initial values could be provided, in principle, by an expert physician.

Numerical estimation of parameters *k*, *μ*_*e*_, *p*, *μ*_*V*_, and of the initial viral load *V*_0_, were carried out using DIFFPAR, a numerical tool written in MATLAB [28, 29]. And consequently, the numerical evaluation of the threshold parameter *R*_0_ is directly obtained. To this purpose, numerical viral loads are generated by solving numerically the model (5) for a 30-day period, for a given parameter vector *θ* and initial conditions. At 10% normal distributed noise simulating measurement errors are added to this data. Next, two circumstances are presented.

1. Initial conditions are known exactly for all the variables.
2. The exact initial value is known only for *H*_*s*0_ = 4500, and *H*_*e*0_ = 500. In this case, a 10% normal distributed noise is added to the initial load *V*_0_. Then parameters and initial viral load are estimated.

The parameter estimation and initial conditions are determined applying the classical least square criterion, or thus, minimizing with respect to *θ* and *η* the objective function

(29) $$\begin{array}{c} g\left(\theta ,\eta \right)=\frac{1}{2}\overset{m}{\underset{i=0}{\sum }}w_{i}{\left(V_{i}-V\left(t_{i};t_{0},\eta ,\theta \right)\right)}^{2}, \end{array}$$

where *η* = *V*_0_, *θ* = (*k*, *μ*_*e*_, *p*, *μ*_*V*_), and *V*(*t*; *t*_0_, *η*, *θ*) is the solution for viral load in reduced model (5). The weights *w*_*i*_ were calculated according to the following rules:

(30) $$w_{i}=\{\begin{array}{cc} \frac{1}{V_{i}^{2}}, & \text{if}\;V_{i}>\sqrt{u},\\ 0, & \text{if}\;V_{i}\leq \sqrt{u}, \end{array}$$

where *u* is the rounding unit in the IEEE standards for double precision floating point arithmetic.

### 6.1. Cured Case

Data was generated using parameters

(31) $$\begin{array}{cc} \theta^{*} & \equiv \left(\beta_{s}^{*},k^{*},\mu_{s}^{*},\mu_{e}^{*},p^{*},\mu_{V}^{*}\right)\\ & =\left(100,\;3\times 10^{-5},\;2\times 10^{-2},\;5,\;100,\;5\right) \end{array}$$

and initial conditions

(32) $$\begin{array}{cc} y_{0} & \equiv \left(H_{s0},H_{e0},V_{0}\right)\\ & =\left(4500,\;500,\;400\right). \end{array}$$

In this case, *R*_0_ = 0.6. The data was generated using initial viral load *V*_0_ with and without noise (see Table 1).

Using only viral load data (*V*), second column in Table 1, and initial conditions (32), we estimate the parameters *k*, *μ*_*e*_, *p*, and *μ*_*V*_. The optimization process began with the initial values for parameters

(33) $$\begin{array}{c} \theta_{0}=\left(100,\;9\times 10^{-5},\;2\times 10^{-2},\;2,\;70,\;10\right). \end{array}$$

**Figure 8:**
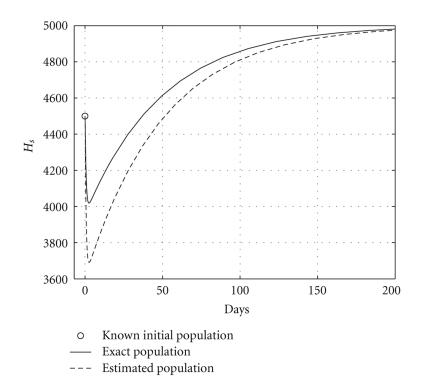
Cured case: comparing the health hepatocyte populations, *H*_*s*0_, *H*_*e*0_, and *V*_0_ are given.

**Figure 9:**
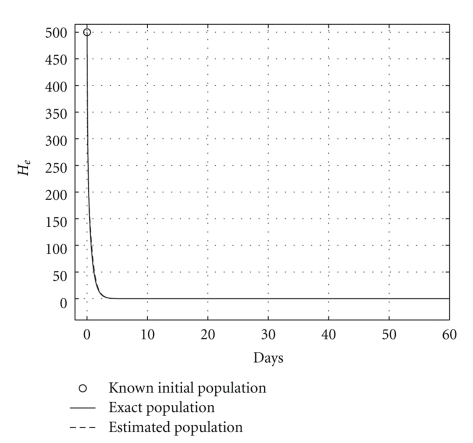
Cured case: comparing the infected hepatocyte populations (hepatic damage), *H*_*s*0_, *H*_*e*0_, and *V*_0_ are given.

**Figure 10:**
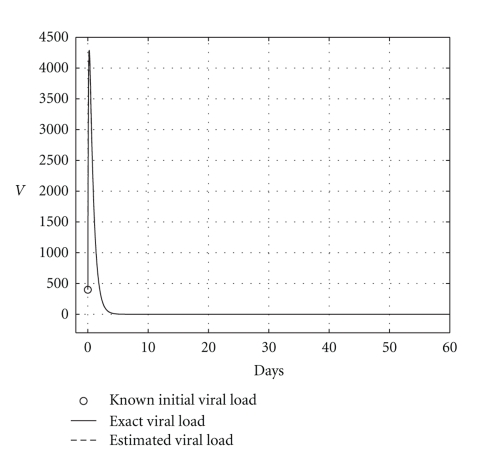
Cured case: comparing the viral loads, *H*_*s*0_, *H*_*e*0_, and *V*_0_ are given.

Table 2 shows the results obtained using DIFFPAR two times. The temporal courses of the populations are shown in Figures 8, 9, and 10. Observe that in Figures 9 and 10 theoretical and estimated curves are practically the same.

The 95% confidence interval for each parameter of Table 2 are given by following inequalities (estimated parameters appear in the middle):

(34) $$\begin{array}{cc} 5.1345\times 10^{-5} & \leq 5.1354\times 10^{-5}\leq 5.1362\times 10^{-5},\\ 6.3933 & \leq 6.3993\leq 6.4053,\\ 123.90 & \leq 123.91\leq 123.93,\\ 6.3999 & \leq 6.4062\leq 6.4125. \end{array}$$

In an analogous way, using only viral loads data in Table 1, to estimate *k*, *μ*_*e*_, *p*, and *μ*_*V*_, and initial viral load *V*_0_, the initial values for optimization process was *V*_0_ = 700, and

(35) $$\begin{array}{c} \theta_{0}=\left(100,\;9\times 10^{-5},\;2\times 10^{-2},\;2,\;70,\;10\right). \end{array}$$

**Figure 11:**
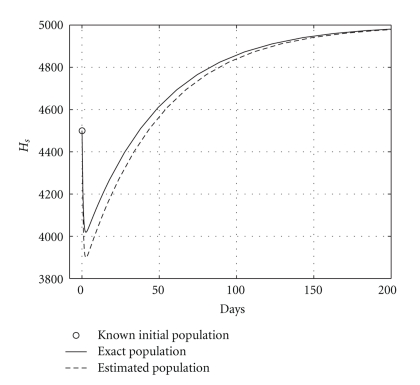
Cured case: comparing the health hepatocyte populations, *H*_*s*0_ and *H*_*e*0_ are known.

**Figure 12:**
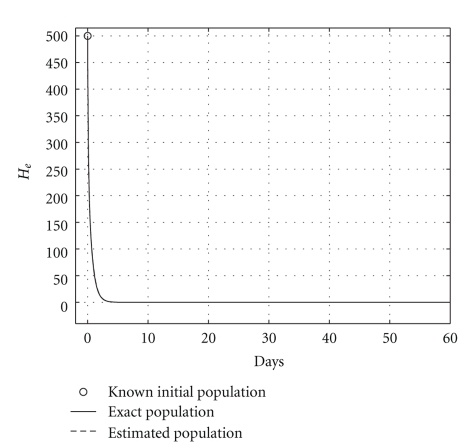
Cured case: comparing the infected hepatocyte populations, *H*_*s*0_ and *H*_*e*0_ are known.

**Figure 13:**
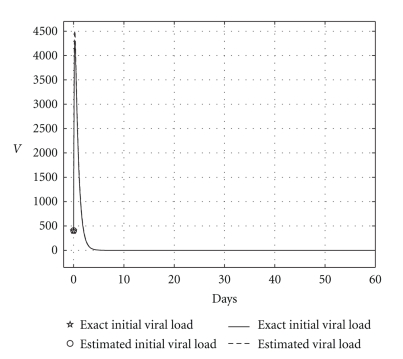
Cured case: comparing the viral loads, *H*_*s*0_, and *H*_*e*0_ are known.

The results are presented in Table 3. Health and infected hepatocyte populations and viral load evolutions are shown in Figures 11, 12, and 13. The estimated parameters and their 95% confidence intervals are

(36) $$\begin{array}{cc} 3.5931\times 10^{-5} & \leq 3.5940\times 10^{-5}\leq 3.5949\times 10^{-5},\\ 5.5961 & \leq 5.6029\leq 5.6097,\\ 115.44 & \leq 115.46\leq 115.48,\\ 5.4937 & \leq 5.5006\leq 5.5076,\\ 404.74 & \leq 405.17\leq 405.60. \end{array}$$

### 6.2. Endemic Case

**Figure 14:**
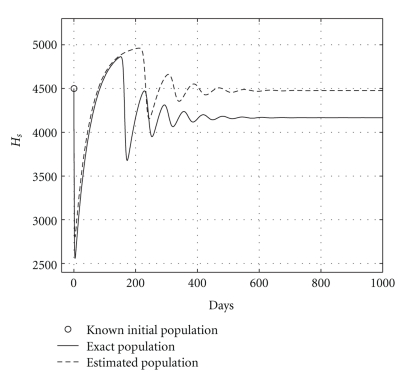
Endemic case: comparing healthy hepatocyte populations, *H*_*s*0_, *H*_*e*0_, and *V*_0_ known.

**Figure 15:**
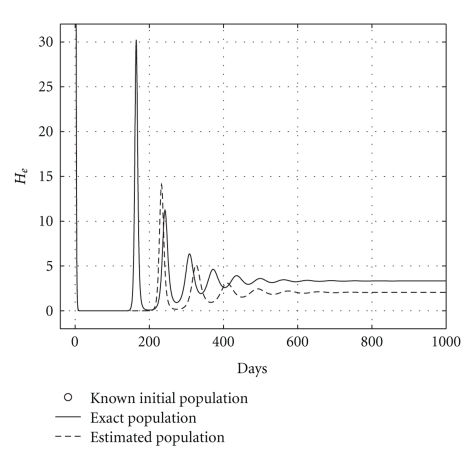
Endemic case: comparing infected hepatocyte populations (hepatic damage) when *H*_*s*0_, *H*_*e*0_, and *V*_0_ are known.

**Figure 16:**
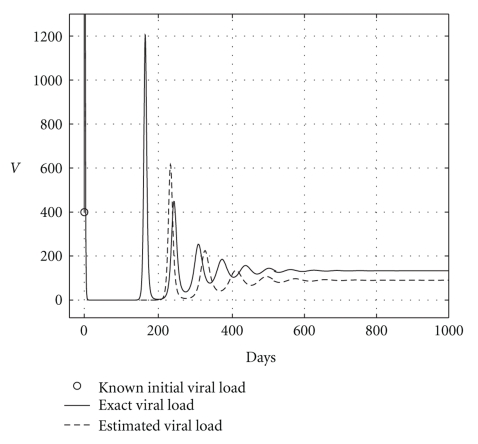
Endemic case: comparing viral load evolutions when *H*_*s*0_, *H*_*e*0_, and *V*_0_ are known.

**Figure 17:**
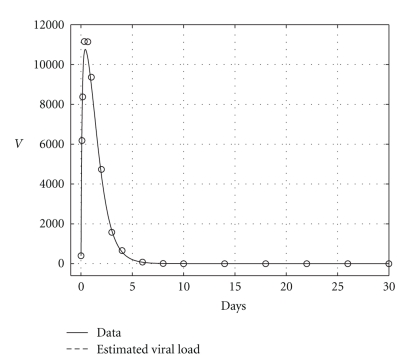
Endemic case: comparing viral data, and estimated viral load evolution when *H*_*s*0_, *H*_*e*0_, and *V*_0_ are known.

Here, data was generated using the same parameter vector θ* given in (31), with exception of p*, whose value was 200. Also the same initial conditions (32) were used. In this case, *R*_0_ = 1.2. As previously mentioned, data was generated using initial viral load *V*_0_ with and without noise (see Table 4). To estimate the parameters, the optimization process began with the initial values for them

(37) $$\begin{array}{c} \theta_{0}=\left(100,\;9\times 10^{-5},\;2\times 10^{-2},\;3,\;300,\;10\right) \end{array}$$

and the results are shown in Table 5. Health and infected hepatocyte populations and viral load evolutions are shown in Figures 14, 15, 16, and 17.

Estimated parameters and its 95% confidence intervals are the following:

(38) $$\begin{array}{cc} 2.5953\times 10^{-5} & \leq 2.5954\times 10^{-5}\leq 2.5955\times 10^{-5},\\ 5.0848 & \leq 5.0867\leq 5.0885,\\ 221.37 & \leq 221.38\leq 221.38,\\ 5.0544 & \leq 5.0563\leq 5.0581. \end{array}$$

Now, using only viral loads (*V*), third column in Table 4, we estimate the parameters *k*, *μ*_*e*_, *p*, and *μ*_*V*_, and initial viral load *V*_0_. The initial values for optimization process were *V*_0_ = 700 and

(39) $$\begin{array}{c} \theta_{0}=\left(100,\;10^{-4},\;2\times 10^{-2},\;15,\;500,\;30\right). \end{array}$$

The results are presented in Table 6. Estimated parameters, and its 95% confidence intervals are the following:

(40) $$\begin{array}{cc} 3.3347\times 10^{-5} & \leq 3.3353\times 10^{-5}\leq 3.3359\times 10^{-5},\\ 5.5423 & \leq 5.5508\leq 5.5593,\\ 214.60 & \leq 214.62\leq 214.65,\\ 5.3827 & \leq 5.3910\leq 5.3993,\\ 412.94 & \leq 413.68\leq 414.43. \end{array}$$

**Figure 18:**
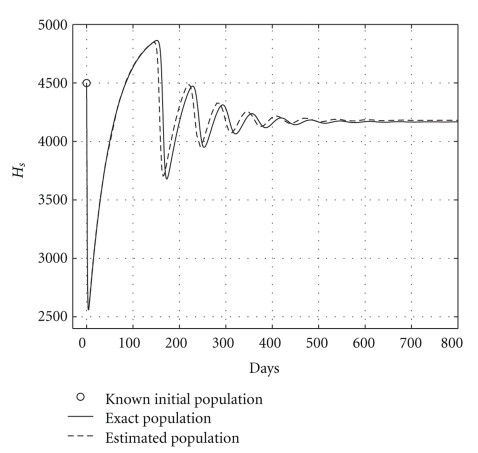
Endemic case: comparing theoretical, and estimated healthy hepatocyte populations when *H*_*s*0_ and *H*_*e*0_ are given.

**Figure 19:**
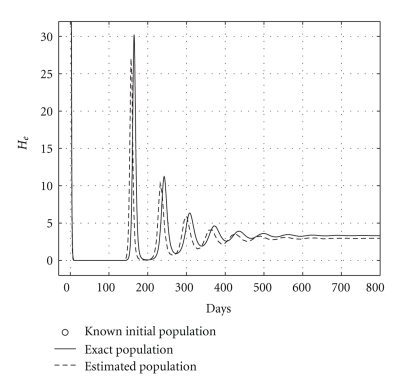
Endemic case: comparing theoretical and estimated infected hepatocyte populations (hepatic damage) when *H*_*s*0_ and *H*_*e*0_ are given.

**Figure 20:**
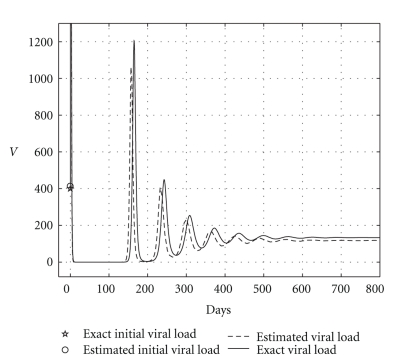
Endemic case: comparing theoretical, and estimated viral load evolutions when *H*_*s*0_ and *H*_*e*0_ are given.

**Figure 21:**
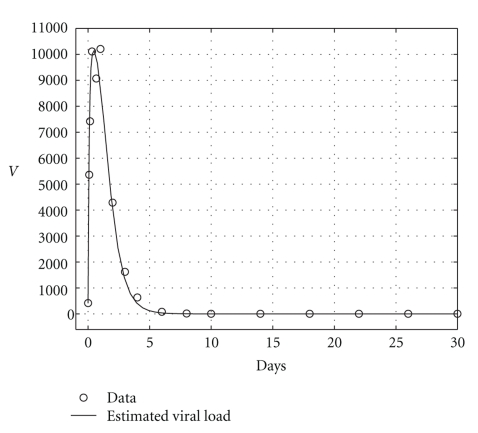
Endemic case: comparing viral data and estimated viral load evolution when *H*_*s*0_ and *H*_*e*0_ are given.

Theoretical and estimated health and infected hepatocyte, and viral load evolutions are shown in Figures 18, 19, 20, and 21.

## 7. Discussion

Firstly, we have shown that qualitative behavior of hepatitis C disease evolution by using the reduced model of three populations (health and infected hepatocytes, and viral load) is essentially the same that the obtained by using the original model proposed in [11] (Sections 3 and 4). Although, some differences are observed in the endemic case, they have eventually the same behavior (Section 5).

As second conclusion, we have that theoretical and estimated disease evolutions of cured cases by using the reduced model, eventually will be observed practically the same behavior. Even though, in the endemic case some evolution differences could be noted, the threshold parameter estimation is good enough.

The third one is our main conclusion. This consists in to showing that, with the reduced model help and carrying out numerical simulations, it is completely feasible to warrant the hepatic damage monitoring without biopsies, under the assumption that we have sufficient number of viral load measurements (from a statistical point of view, to have two or three viral loads measurements per each parameter to be estimated, in our case, from 10 to 15), and reasonable good initial estimation of the amount of hepatic tissue damaged, supplied by an expert clinician and hepatopathologist. It is very important to say that this monitoring procedure does not have any inconvenience (reliability, complication risks, patient pain, and other negative aspects), as occurs with conventional biopsies. Even when today percutaneous biopsy for diagnosing hepatic damage is not so important, the hepatic damage monitoring is a worthy tool in addition to the viral load, making possible a good followup. So, even we have not yet any experimental research with patients in order to model calibration and validation, our proposal for monitoring viral load and hepatic damage evolution (Section 6) represents an innovative new, worthy, and reliable tool to carry out for hepatitis C disease tracking, taking account of genotypes and ethnic considerations [3]. This fact provides a theoretical foundation to the protocol nowadays used for hepatitis C treatment without biopsies.

## Figures and Tables

**Table 1:** Generated data for cured case (*R*_0_ = 0.6).

	Viral load (*V*)	Viral load (*V*)
Time (*t*)	(UI/*μ*L)	(UI/*μ*L)
	(*V*_0_ without noise)	(*V*_0_ with noise)
0 hours	400	4.0501329 × 10^2^
2 hours	3.1826295 × 10^3^	3.4311542 × 10^3^
4 hours	4.0389101 × 10^3^	4.1913114 × 10^3^
8 hours	4.5737159 × 10^3^	4.4215144 × 10^3^
16 hours	3.0550250 × 10^3^	2.7656531 × 10^3^
24 hours	1.7487986 × 10^3^	1.7521023 × 10^3^
2 days	3.7767239 × 10^2^	3.9899151 × 10^2^
3 days	7.1813740 × 10^1^	9.7917259 × 10^1^
4 days	1.8916925 × 10^1^	2.0639365 × 10^1^
6 days	9.4351220 × 10^−1^	9.3888612 × 10^−1^
8 days	3.9986367 × 10^−2^	4.8010863 × 10^−2^
10 days	2.0585181 × 10^−3^	2.4422741 × 10^−3^
14 days	7.5294784 × 10^−6^	7.6147111 × 10^−6^
18 days	1.0916324 × 10^−6^	1.1957034 × 10^−6^

**Table 2:** Results when *H*_*s*0_, *H*_*e*0_, and *V*_0_ are known (*R*_0_ = 0.6).

	*k*	*μ*_*e*_	*p*	*μ*_*V*_	*R*_0_
θ* Exact	3 × 10^−5^	5	100	5	0.6
$\hat{\theta }$ Estimated	5.1354 × 10^−5^	6.3993	123.91	6.4062	0.78
Error	71.18%	27.99%	23.91%	28.12%	30.00%

**Table 3:** Results when *H*_*s*0_ and *H*_*e*0_ are known (*R*_0_ = 0.6).

	*k*	*μ*_*e*_	*p*	*μ*_*V*_	*V*_0_	*R*_0_
θ* Exact	3 × 10^−5^	5	100	5	400	0.6
$\hat{\theta }$ Estimated	3.5940 × 10^−5^	5.6029	115.46	5.5006	405.17	0.67
Error	19.80%	12.06%	15.46%	10.01%	1.29%	11.7%

**Table 4:** Generated data for endemic case (*R*_0_ = 1.2).

	Viral load (*V*)	Viral load (*V*)
Time (*t*)	(UI/*μ*L)	(UI/*μ*L)
	(*V*_0_ without noise)	(*V*_0_ with noise)
0 hours	400	4.1309470 × 10^2^
2 hours	6.1816864 × 10^3^	5.3573206 × 10^3^
4 hours	8.3762150 × 10^3^	7.4186646 × 10^3^
8 hours	1.1150813 × 10^4^	1.0106325 × 10^4^
16 hours	1.1135837 × 10^4^	9.0672355 × 10^3^
24 hours	9.3603986 × 10^3^	1.0211713 × 10^4^
2 days	4.7275936 × 10^3^	4.2850412 × 10^3^
3 days	1.5740823 × 10^3^	1.6224563 × 10^3^
4 days	6.5576300 × 10^2^	6.3646394 × 10^2^
6 days	8.0051038 × 10^1^	7.7100090 × 10^1^
8 days	9.0270927 × 10^0^	1.0311416 × 10^1^
10 days	1.3696024 × 10^0^	1.4188935 × 10^0^
14 days	5.0844301 × 10^−2^	4.9818506 × 10^−2^
18 days	2.6024858 × 10^−3^	3.1927516 × 10^−3^
22 days	2.0748529 × 10^−4^	2.2361548 × 10^−4^
26 days	2.6586162 × 10^−5^	2.5486597 × 10^−5^
30 days	5.2690861 × 10^−6^	7.0851754 × 10^−6^

**Table 5:** Results when *H*_*s*0_, *H*_*e*0_, and *V*_0_ are known (*R*_0_ = 1.2).

	*k*	*μ*_*e*_	*p*	*μ*_*V*_	*R*_0_
θ* Exact	3 × 10^−5^	5	200	5	1.2
$\hat{\theta }$ Estimated	2.5954 × 10^−5^	5.0867	221.38	5.0563	1.117
Error	13.49%	1.73%	10.69%	1.13%	6.92%

**Table 6:** Results when *H*_*s*0_ and *H*_*e*0_ are given (*R*_0_ = 1.2).

	*k*	*μ*_*e*_	*p*	*μ*_*V*_	*V*_0_	*R*_0_
θ* Exact	3 × 10^−5^	5	200	5	400	1.2
$\hat{\theta }$ Estimated	3.3353 × 10^−5^	5.5508	214.62	5.3910	413.68	1.1961
Error	11.2%	11.02%	7.3%	7.8%	3.4%	0.33%
